# Validation of the revised 9-scale clinical frailty scale (CFS) in Greek language

**DOI:** 10.1186/s12877-021-02318-3

**Published:** 2021-06-29

**Authors:** Ioannis Vrettos, Panagiota Voukelatou, Stefani Panayiotou, Andreas Kyvetos, Andreas Kalliakmanis, Konstantinos Makrilakis, Petros P. Sfikakis, Dimitris Niakas

**Affiliations:** 1 2nd Department of Internal Medicine, General and Oncology Hospital of Kifissia “Agioi Anargyroi”, Noufaron and 14 Timiou Stavrou street, Athens, Greece; 2 Department of Health Economics, School of Health Sciences, National and Kapodistrian University of Athens, 75 Mikras Asias street, Athens, Greece; 3 1st Department of Propedeutic Internal Medicine, Laikon General Hospital, 17 Agiou Thoma street, Athens, Greece; 4 School of Medicine, National and Kapodistrian University of Athens, 75 Mikras Asias street, Athens, Greece; 5 1st Department of Propedeutic Internal Medicine-Rheumatology Unit, Laikon General Hospital, 17 Agiou Thoma street, Athens, Greece

**Keywords:** Frailty, Clinical frailty scale, Elderly, Validation

## Abstract

**Background:**

Among many screening tools that have been developed to detect frailty in older adults, Clinical Frailty Scale (CFS) is a valid, reliable and easy-to-use tool that has been translated in several languages. The aim of this study was to develop a valid and reliable version of the CFS to the Greek language.

**Methods:**

A Greek version was obtained by translation (English to Greek) and back translation (Greek to English). The “known-group” construct validity of the CFS was determined by using test for trends. Criterion concurrent validity was assessed by evaluating the extent that CFS relates to Barthel Index, using Pearson’s correlation coefficient. Both inter-rater and test–retest reliability were assessed using intraclass correlation coefficient.

**Results:**

Known groups comparison supports the construct validity of the CFS. The strong negative correlation between CFS and Barthel Index (r_s_ = − 0,725, *p* ≤ 0.001), supports the criterion concurrent validity of the instrument. The intraclass correlation was good for both inter-rater (0.87, 95%CI: 0.82–0.90) and test-retest reliability (0.89: 95%CI: 0.85–0.92).

**Conclusion:**

The Greek version of the CFS is a valid and reliable instrument for the identification of frailty in the Greek population.

## Background

Older adults are a highly heterogeneous group, with differences in their health and functional status. Consequently, people with the same chronological age can have different biological ages [1]. In the last 30 years the term *frailty* is used more and more [2] to understand and describe the health diversity among them. Frailty is conceptualized as the result of the aging process that leads to cumulative decline in many physiological systems and to increased risk of vulnerability [3]. According to the definition of a consensus group, consisting of delegates from six major international, European, and US societies, frailty is “a medical syndrome with multiple causes and contributors that is characterized by diminished strength, endurance, and reduced physiologic function that increases an individual’s vulnerability for developing increased dependency and/or death” [4].

Among many screening tools that have been developed to detect frailty in older adults [5] Clinical Frailty Scale (CFS) is a valid, reliable and easy-to-use tool that allows health-care providers to assign a score based only on a standard clinical interview [6], and can also be reliably used retrospectively [7]. It has been introduced as a seven-point scale, ranging from very fit to severely frail, with a visual chart that accompanied a description for each point of the scale [6]. Later, it was expanded from a 7-point scale to the present 9-point scale [8] and recently was further revised with minor edits to the level descriptions and their corresponding labels [9]. It has been largely used to assess the overall level of fitness or frailty in hospitalized [10–14], institutionalized [15–17] and community-dwelling [6, 7] older adults and in elderly patients admitted to intensive care units [18–20] or evaluated at emergency departments [21–23].

As frailty has been associated with mortality [6, 10, 11], length of hospitalization [24–26], degree and time of recovery [12, 27], re-admission [11, 25, 28], and future need for institutionalization [6, 24, 29], there is a need for tools that can be used practically and quickly to detect frailty [30]. In order to avoid misclassification due to differences in culture or how someone perceives the English version individually [31], CFS has been translated in several languages [30–36]. Trying to promote the adequate use of this scale in Greece we aimed to develop a valid and reliable version of the CFS to the Greek language.

## Methods

### Sample, tools and data collection

A prospective study was conducted among patients older than 65 years old, consecutively admitted through the emergency department of General and Oncological Hospital of Kifissia “Agioi Anargyroi” from September 2020 to January 2021. On admission, after a comprehensive geriatric assessment (CGA) that requires the evaluation of physical, cognitive, affective, social, financial, and environmental components [37], patients’ demographic characteristics (age, gender, educational level, marital status), medical history (comorbidities), medication use (number and type of medications) and reason of admission were recorded.

Charlson Co-morbidity Index (CCI), which includes most major medical comorbidities [38], was used, for measuring co-morbidity, while activities of daily living were evaluated using Barthel Index [39]. Cognitive status was assessed by using the Global Deterioration Scale, a 7-point scale ranging from no cognitive decline (stage 1) to very severe cognitive decline -severe dementia (stage 7) that can be broken down into three groups (no cognitive decline, mild cognitive impairment, and severe- very severe cognitive impairment) [40]. Both Barthel Index and Global Deterioration Scale were estimated for the baseline status of the patients, when not affected by acute illness. Information regarding demographic characteristics, medical and medication history and functional status were obtained by asking either the patients or their caregivers, when patients were not able to communicate.

After the initial assessment, CFS was scored for each patient (CFS_1_). In order to evaluate inter-rater reliability, a second CFS assessment was performed by another examiner who did not know the other’s score (CFS_2_). CFS was also re-assessed by the initial examiner, to evaluate test-retest reliability, at least 2 weeks later, after interrogation of the entire patients’ record (CFS_3_). CFS_1_, CFS_2_ and CFS_3_ were scored according to the baseline function of the patient, before the onset of acute illness precipitating hospital admission. Before starting this study, the two examiners underwent training regarding the assessment of frailty by using CFS.

The research protocol was approved by the institutional ethical and scientific committee. An informed written consent was obtained from the patients or from their family members.

### Obtaining the Greek version of CFS

**Fig. 1:**
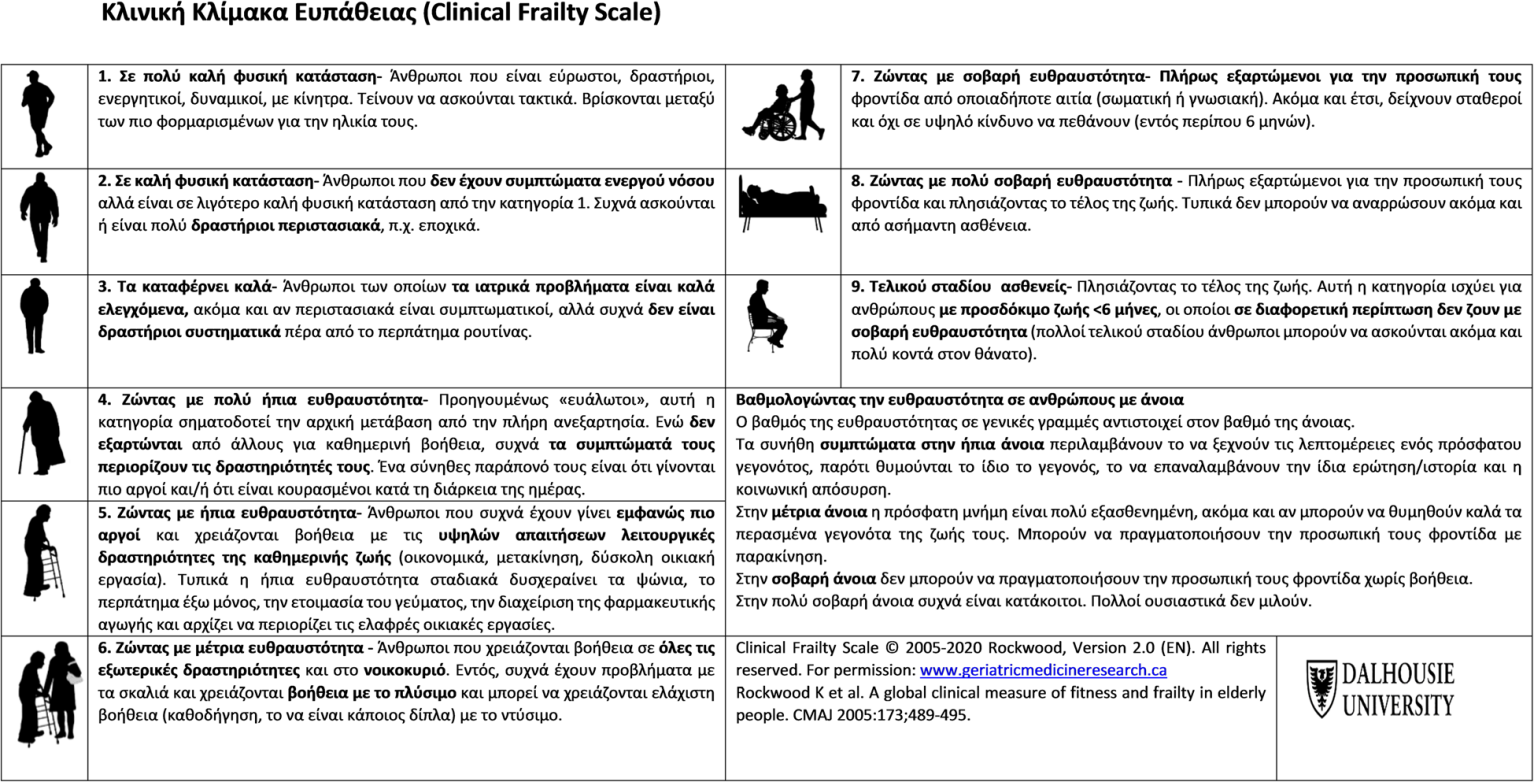
Clinical Frailty Scale in Greek language

After obtaining permission from the original authors, two independent translations of the Clinical Frailty Scale, from English into Greek, were done by a translation agency and by a medical doctor with certified excellent knowledge of the English language. The two versions were compared and a consensus-based choice of an appropriate translation was performed by the authors. Then, the Greek version of CFS was retranslated into English by a professional translator and a doctor whose native language was Greek and lives in England. The two back-translators were blinded to the original questionnaire. The authors compared the two back-translated versions with the original and the differences were resolved by agreement between the authors, aiming to improve the Greek translated version. The Greek version was then further assessed by six medical doctors whose native language is Greek and their comments were used to further modify the scale and obtain the definite Greek version (Fig. 1).

### Validity and reliability of the Greek version of CFS

The “known-group” construct validity of the CFS was determined by examining hypothesized relationships between sociodemographic and health-related variables and the level of fitness or frailty according to CFS. Specifically, it was expected that the presence of frailty would be associated with older age, higher CCI, mobility problems, falls in previous months, social withdrawal, swallowing problems and the degree of cognitive impairment.

Criterion concurrent validity was assessed by examining the association between CFS and Barthel Index.

CFS_1_ and CFS_2_ scores were used for the evaluation of inter-rater, and CFS_1_ and CFS_3_ scores were used for the evaluation of test-retest reliability respectively.

### Statistical analysis

All analyses were performed by using SPSS v22.0. For assessing the distribution of evaluated continuous variables the Kolmogorov–Smirnov test was used. The continuous variables: age, CCI, and number of medications had non-Gaussian distribution and are expressed as median and interquartile range. Categorical variables are expressed as percentages. Patients who were scored 1–3 at CFS were grouped as non-frail and patients who were scored ≥4 were grouped as frail. Construct validity was evaluated by using known groups comparison to test how well the CFS discriminates between subgroups of the study sample that differed in age, CCI, mobility, balance, sociability, swallowing ability and the degree of cognitive impairment. Test for trends was used for comparisons. When p level was < 0.05 the results were considered statistically significant. Criterion concurrent validity was assessed evaluating the extent that CFS relates to Barthel Index, using Pearson’s correlation coefficient. Both inter-rater and test–retest reliability of CFS were assessed by using intraclass correlation coefficient with 95% confidence intervals (CI).

## Results

During the study period, 145 older patients were admitted to the medical unit through the emergency department. Two of them (one man and one woman) were reluctant to participate and for one more, who was unable to communicate, his caregiver denied to participate in the study. The median age of patients was 82.00 (IQR: 75.75-87.00). Among the participants 74 were women (52.1%) and 68 men (47.9%). As frail were categorized 87 patients (61.3%). Patients’ characteristics are presented in Table 1.

**Table 1:** Patients’ characteristics

	*n* = 142
**Gender**	
Males	68 (47.9%)
Females	74 (52.1%)
**Age** (median-IQR) (years old)	82.00 (75.75-87.00)
**CCI** (median-IQR)	5.00 (4.00-7.00)
**Number of medications** (median-IQR)	5.50 (3.00-7.25)
**Marital status**	
Married	74 (52.1%)
Unmarried	2 (1.4%)
Divorced	5 (3.5%)
Widowed	61 (43.0%)
**Educational status**	
Primary	70 (49.3%)
Secondary	49 (34.5%)
Technological Education Institution	11 (7.7%)
University	12 (8.5%)
**Living alone**	
Yes	19 (13.4%)
No	123 (86.6%)
**Barthel index groups**	
No dependency (BI ≥95)	53 (37.3%)
Mild-moderate dependency (BI 90–65)	46 (32.4%)
Moderate-severe dependency (BI 60–25)	25 (17.6%)
Absolute dependency (BI ≤20)	18 (12.7%)
**Degree of cognitive impairment**	
No cognitive impairment	88 (62.0%)
Mild-moderate cognitive impairment (equivalent to GDS ≤ 5)	36 (25.4%)
Severe-very severe cognitive impairment (equivalent to GDS ≥ 6)	18 (12.7%)
**CFS groups**	
Frail	87 (61.3%)
Non frail	55 (38.7%)

*IQR* Interquartile Range, *CCI* Charlson Co-morbidity Index, *GDS* Global Deterioration Scale, *CFS* Clinical Frailty Scale

**Fig. 2:**
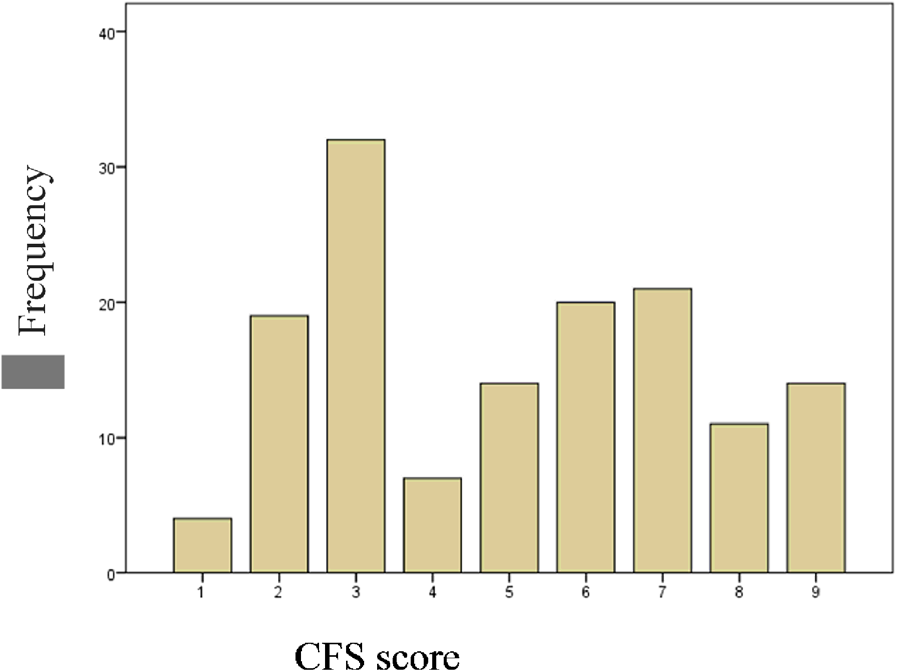
Number of patients across different CSF scores

The more prevalent CFS phenotype was 3-“Managing Well” (32 patients), followed by 7-“Living with Severe Frailty” (21 patients) and 6-“Living with Moderate Frailty” (20 patients). The distribution of patients across different CSF scores is illustrated in Fig. 2.

**Fig. 3:**
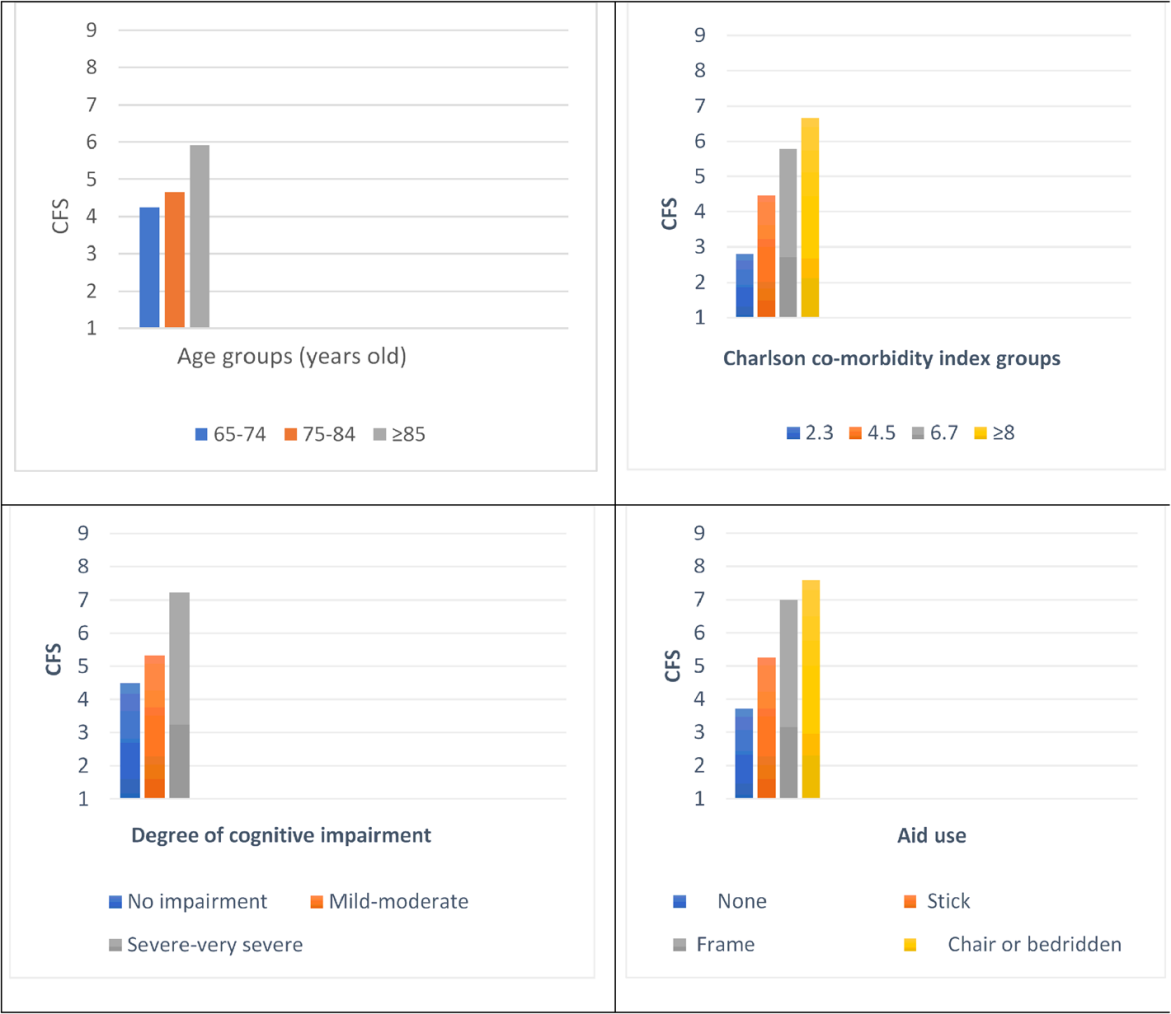
CFS scores across subgroups of elderly based on age, Charlson Co-morbidity Index, degree of cognitive impairment and aid use

Known groups comparison showed that CFS discriminated well between subgroups of people who were differed in age, CCI, mobility, balance, sociability, swallowing ability and the degree of cognitive impairment. As hypothesized the oldest old, those with affected mobility, balance and swallowing ability and respondents who were socially withdrawn or had impaired cognitive status, had higher CFS scores. The differences in CFS scores across the subgroups were statistically significant and confirmed expected relationships, supporting the construct validity of the instrument (Table 2 and Fig. 3).

**Table 2:** CFS scores across subgroups of elderly, categorized according to socio-demographic and health related characteristics

Socio-demographic and health related characteristics	n	CFS score (Μ ± 1SD)	Statistical significance^*^
**Age groups (years old)**			
65-74	29	4.24 ± 2.60	*p* = 0.002
75-84	59	4.66 ± 2.40	
≥ 85	54	5.91 ± 1.96	
**Charlson Co-morbidity Index groups**			
2-3	15	2.80 ± 1.74	*p* ≤ 0.001
4-5	62	4.47 ± 2.09	
6-7	41	5.80 ± 1.96	
≥ 8	24	6.67 ± 2.55	
**Aid use**			
None	74	3.72 ± 2.04	*p* ≤ 0.001
Stick	27	5.26 ± 1.91	
Frame	19	7.00 ± 1.49	
Chair or bedridden	22	7.59 ± 0.67	
**Falls in previous months**			
No	99	4.40 ± 2.39	*p* ≤ 0.001
Yes	43	6.53 ± 1.53	
**Socially engaged**			
Frequent	49	3.43 ± 2.28	*p* ≤ 0.001
Occasional	59	5.49 ± 1.88	
Not	34	6.62 ± 1.89	
**Swallowing problems**			
No	121	4.63 ± 1.91	*p* ≤ 0.001
Yes	21	7.48 ± 0.88	
**Degree of cognitive impairment**			
No cognitive impairment	88	4.49 ± 2.44	*p* ≤ 0.001
Mild-moderate cognitive impairment	36	5.33 ± 2.07	
Severe-very severe cognitive impairment	18	7.22 ± 0.88	

^*^Derived from test for trends

Pearson’s correlation coefficient (r) was applied to measure the association between CFS and Barthel Index. There was a strong negative correlation among them, which was statistically significant (r_s_ = − 0,725, *p* ≤ 0.001), supporting the criterion concurrent validity of the instrument. The intraclass correlation was good both for inter-rater reliability, being 0.87 (95%CI: 0.82–0.90) and also for test-retest reliability, being 0.89 (95%CI: 0.85–0.92).

## Discussion

The main aim of this study was to translate in the Greek language and validate the revised nine-scale CFS instrument for the evaluation of frailty in elderly patients with a simple and quick way, offering a suitable instrument to the Greek scientific community to identify frailty. In our study, elderly patients were categorized in two groups: frail and non-frail according to the revised version of CFS in which the level description 4 changed from “Vulnerable” to “Living with very mild frailty” [9]. Therefore, patients who were categorized as level 4 were counted as frail. Otherwise, before the revision of the CFS, patients would be classified in three groups [8]: frail (80 patients, 56.4%), vulnerable (7 patients, 4.9%) and non-frail (55 patients, 38.7%).

In this study, we showed that CFS was able to distinguish between groups of elderly patients in the expected manner (known-groups validity) on the basis of age, CCI, mobility, balance, sociability, swallowing ability and the degree of cognitive impairment, providing evidence of its construct validity.

Moreover, the strong negative correlation between CFS and Barthel Index supports the criterion concurrent validity of the instrument. Barthel Index, is an ordinal scale, used to assess performance in activities of daily living [39] and not a direct measure of frailty. Nevertheless, activities of daily living are an essential component of frailty [6, 41, 42] and frailty is related directly with disability in activities of daily living [43]. Therefore, the correlation between CFS and Barthel Index is in line with Taherdoost’s [44] definition, that defines criterion concurrent validity as “the extend that a measure simultaneously relates to another measure that it is supposed to relate”.

Regarding the reliability of CFS, overall, the Greek version exhibited good inter-rater and test-retest reliability.

In Greece only a few studies have been conducted concerning frailty. CFS has only been used twice for research purposes. In the first study, CFS was used evaluating frailty in older patients admitted in an intensive care unit. In this study 25% of the patients were categorized as frail, based on information adapted by the patients’ family or caregiver [45]. In the second one, CFS was used to assess the frailty status of hospitalized elderly patients with atrial fibrillation. Frailty status was found to affect decisions regarding long term anticoagulation therapy [46]. Translation and validation of CFS was not mentioned in both of these studies. The same applies to other studies referring to frailty in patients suffering from multiple myeloma [47] or chronic obstructive pulmonary disease [48] and in older Greek women [49, 50], where different tools, rather than CFS, were used. Only recently, Tilburg Frailty Indicator was translated and validated in Greek language in a sample of older patients attending an Urban Health Center [51].

Taking into consideration that in Greece there is a lack of translated and validated frailty screening tools such as CFS, that can be applied in multiple settings [52], it is clear that the Greek version may promote the evaluation of frailty in the Greek population, improving patients’ quality of care and outcomes. More specifically, frailty assessment by using Greek CFS can be applied to guide older patients’ care, taking into consideration the probable risks and benefits, to provide individualized care and to identify those at risk for negative health consequences [52]. Furthermore, the early identification of frailty may guide interventions in order to prevent or reverse disability in older persons [53]. However, at this time, despite some efforts to apply frailty assessment into health-care policy [4, 54, 55] and despite the numerous studies dealing with frailty, the need for the application of all this knowledge into clinical practice still exists [1, 56, 57].

The main study limitation is the lack of a validated Greek translation of another screening tool for the identification of frailty, to compare it with the Greek version of CFS, as a reference method, in order to evaluate its concurrent validity. As mentioned before, the only valid translated tool for frailty assessment, available in Greek language is Tilburg Frailty Indicator [51]. However, this tool includes only self-reported information and it has been developed for the assessment of frailty in the community [58]. So, its use was inappropriate for our study population. Another limitation is that the study sample consisted of hospitalized patients and so, results regarding the prevalence of frailty or other study sample characteristics cannot be generalized in a community-based population.

## Conclusion

Τhe results of our study demonstrated that the Greek version of the revised nine-scale CFS is a valid and reliable instrument for the identification of frailty in Greek population.

## Data Availability

The datasets used and/or analyzed during the current study are available from the corresponding author on reasonable request.

## Abbreviations

CFS: Clinical Frailty Scale;
CCI: Charlson Co-morbidity Index
GDS: Global Deterioration Scale
SPSS: Statistical Package for the Social Sciences
